# Multi-terminal Josephson junctions as topological matter

**DOI:** 10.1038/ncomms11167

**Published:** 2016-04-04

**Authors:** Roman-Pascal Riwar, Manuel Houzet, Julia S. Meyer, Yuli V. Nazarov

**Affiliations:** 1 University of Grenoble Alpes, INAC-PHELIQS, F-38000 Grenoble, France; 2 CEA, INAC-PHELIQS, F-38000 Grenoble, France; 3 Kavli Institute of NanoScience, Delft University of Technology, Lorentzweg 1, Delft NL-2628 CJ, The Netherlands

## Abstract

Topological materials and their unusual transport properties are now at the focus of modern experimental and theoretical research. Their topological properties arise from the bandstructure determined by the atomic composition of a material and as such are difficult to tune and naturally restricted to ≤3 dimensions. Here we demonstrate that *n*-terminal Josephson junctions with conventional superconductors may provide novel realizations of topology in *n*−1 dimensions, which have similarities, but also marked differences with existing 2D or 3D topological materials. For *n*≥4, the Andreev subgap spectrum of the junction can accommodate Weyl singularities in the space of the *n*−1 independent superconducting phases, which play the role of bandstructure quasimomenta. The presence of these Weyl singularities enables topological transitions that are manifested experimentally as changes of the quantized transconductance between two voltage-biased leads, the quantization unit being 4*e*^2^/*h*, where *e* is the electric charge and *h* is the Planck constant.

## Abstract (web summary)

Materials with topologically nontrivial band structures possess exotic electronic transport properties however they are naturally constrained below three dimensions. Here, the authors demonstrate how analogous systems with *n*−1 dimensions may be constructed from Josephson junctions of *n*-terminals.

## Graphical abstract

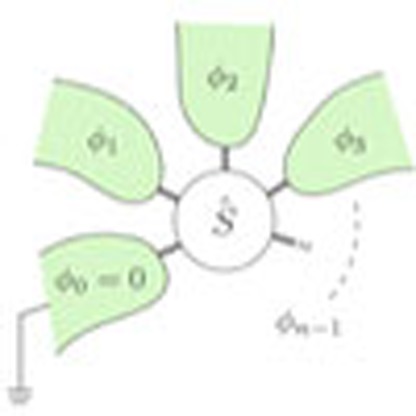

Josephson junctions created by coupling two superconductors through a weak link have been studied extensively for many years^1^,^2^,^3^. The current across a Josephson junction yields information about the Andreev bound states (ABS) forming at the junction^4^,^5^,^6^,^7^. In turn, the ABS spectrum is determined by the properties of the junction and the superconducting leads. For instance, if the leads are topologically nontrivial, the Josephson effect may be used to probe these topological properties. In particular, the 4*π*-periodicity of the supercurrent indicates the presence of topologically protected zero-energy Majorana states^8^,^9^,^10^,^11^, which may arise in one-dimensional spinless p-wave superconductors, semiconductor nanowires with proximity-induced superconductivity or at the surface of bulk materials.

The rapidly growing field of three-dimensional (3D) Weyl semimetals deals with a bandstructure that exhibits conical energy gap closings: Weyl points^12^,^13^,^14^,^15^,^16^. Unlike the Dirac point in graphene^17^ that may be gapped out through an appropriate coupling, isolated Weyl points are topologically protected. They can be regarded as monopoles with a positive or negative charge. A topological invariant—the Chern number—defined on a surface in momentum space characterizes the total charge of the monopoles it encloses. These monopoles give rise to many unusual features, such as chiral edge states and associated surface Fermi arcs^14^. Topological protection guarantees that the only way to induce a gap is either to annihilate two Weyl points of opposite charge by bringing them together, or to couple two cones at a finite distance in momentum space through breaking of momentum conservation^16^.

In this paper, we show that multi-terminal Josephson junctions may be topologically nontrivial even if the superconducting leads are topologically trivial and no exotic materials are used to make the junction. Thus, the junction itself may be regarded as an artificial topological material, which displays Weyl singularities, when the energy of the lowest ABS goes to zero at certain values of the superconducting phases such that the gap in the spectrum closes. Below, we also show that their topological property can be easily probed by the transconductance between two voltage-biased leads, which is proportional to the Chern number.

## Results

### The topology of the bound state spectrum

**Figure 1:**
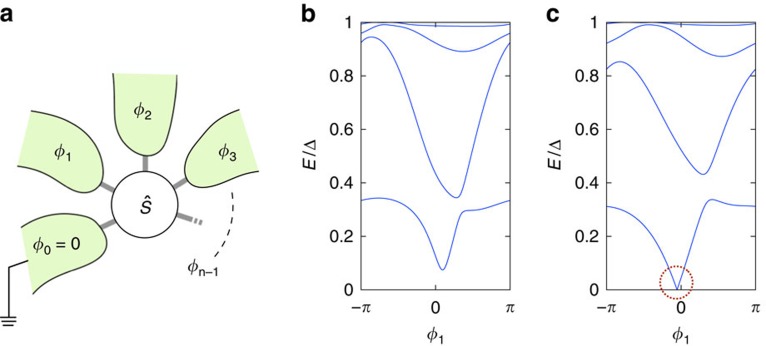
General setup of the multi-terminal junction, and examples of typical ABS spectra. (**a**) The superconducting leads with phases *ϕ*_*α*_, *α*=0, …, *n*−1, are connected through a scattering region described by the scattering matrix 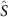. (**b**) Generic ABS energy spectrum versus *ϕ*_1_, away from a Weyl singularity. (**c**) ABS energy spectrum versus *ϕ*_1_, where the other phases are tuned to a Weyl singularity. Note the gap closing (red, dotted circle).

We consider a junction with *n* superconducting leads connected through a scattering region (Fig. 1a). The leads *α*={0, 1, …, *n*−1} have the same gap Δ, though they may differ in the phase of the superconducting order parameter, *ϕ*_*α*_. Due to gauge invariance, only *n*−1 phases are independent, hence we may set *ϕ*_0_=0. Likewise we choose to focus on a short scattering region, characterized by an energy-independent scattering matrix 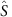 in the basis of *N*=∑_*α*_*N*_*α*_ transport channels, *N*_*α*_ being the number of channels in contact *α*. In a longer scattering region described by an energy-dependent 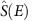, more bound states would appear at finite energy. However, the presence of Weyl singularities at zero energy only depends on 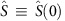. We also assume time-reversal symmetry, such that 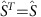, as well as spin-rotation symmetry. Note, although, that our predictions are robust even if those symmetries are broken, see Discussion section.

The junction hosts a set of spin-degenerate ABS, indexed by *k*, whose energies *E*_*k*_≥0 are determined from the equation^4^


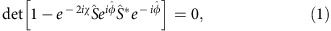


where *χ*=arccos(*E*/Δ), and 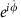 is a diagonal matrix that assigns to each channel the phase factor of the corresponding terminal. The spin-degenerate ABS energies 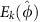 (Fig. 1b) are periodic in all phases with a period 2*π*. The total phase-dependent energy of the junction reads *E*=∑_*kσ*_(*n*_*kσ*_−1/2)*E*_*k*_, *n*_*kσ*_=0, 1 being the occupation of the state *k* with spin *σ*.

Zero-energy states are most easily described by making use of the mapping from states with spin *σ* at energy *E*≥0 to states with spin −*σ* at energy −*E*. Then a gap closing at a certain value of the phases, 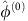, corresponds to the crossing of two (singly degenerate) states. Thus, the zero-energy state is doubly (spin-) degenerate. We can then describe the lowest ABS band in the vicinity of the gap closing by the two-by-two Weyl Hamiltonian (Supplementary Note 1),


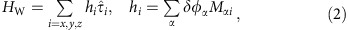


with the Pauli matrices 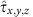 in the basis of the two degenerate states corresponding to eigenvalue *E*=0. The fields *h*_*i*_ depend linearly on 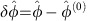 through the real matrix 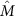.

The form of the Weyl Hamiltonian in equation (2) indicates that we need at least three parameters to tune the system to the degeneracy point, *h*_*i*_=0. Thus, for four terminals with three independent phases, the Weyl singularities appear as points in the 3D phase space. For five terminals, the Weyl singularities occur in general as one-dimensional curves in the four-dimensional (4D) space of phases. This opens up the possibility to realize a topological material in arbitrary dimensions. Note that such multi-terminal Josephson junctions cannot be characterized by means of the standard periodic table of topological semimetals^18^,^19^, due to the distinct behaviour of the quasimomenta 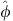 under particle–hole symmetry. A proper classification could be envisioned along the lines of ref. 20.

The topology of the junction can be characterized by a set of Chern numbers. A Chern number may be defined in the two-dimensional (2D) subspace of two phases *ϕ*_*α*_ and *ϕ*_*β*_, through the local Berry curvature of the ABS. The Berry curvature for the bound state *k* with spin *σ* is related to its wave function 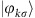 through


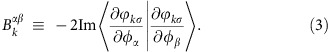


Note that 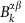 does not depend on spin (Supplementary Note 2). The total Berry curvature of the many-body superconducting state then reads 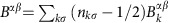, in analogy to the expression for the energy. For fixed occupations *n*_*kσ*_=0, 1, the integral of the Berry curvature over the elementary cell yields an integer, the Chern number


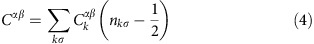


with 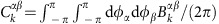.

Since the Weyl singularities appear as points in the 3D phase space, a third phase *ϕ*_*γ*_ may be used to tune the system through Weyl points, thus changing the Chern number. We see that a given band *k* contributes to the total Chern number with 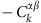 when it is empty, and with 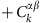 when it is doubly occupied, whereas it gives a zero contribution if there is a single quasiparticle in the band.

### Probing Weyl points through quantized transconductance

Importantly, the current response of the junction with slowly varying phases reveals the Chern number. Biasing lead *β* with a voltage 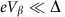 gives rise to the instantaneous current to contact *α* (Supplementary Note 2 and Supplementary Fig. 1)


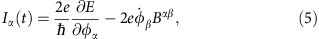


where 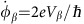. The first term corresponds to the adiabatic current and the second term is the first order correction in the phase velocity. Let us now apply constant voltages to two leads. For incommensurate voltages, the two phases uniformly sweep the elementary cell. In the d.c. limit, the adiabatic current contribution then averages out, and the Berry curvature is replaced by its average value. Thus, we find that the d.c. current is linear in the voltages, and the transconductance is defined by the Chern number


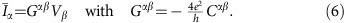


Equation (6) shows that multi-terminal junctions exhibit a d.c. current response typical for the quantum Hall effect, although based on different physics. The transconductance quantum is four times bigger than in the quantum Hall effect, which can be traced to the 2*e* charge of the superconducting Cooper pairs and the presence of two spin bands. To extract the small d.c. signal, the averaging time needs to be sufficiently long. The relevant time scale is determined by the low-frequency current noise (see Supplementary Note 3).

Generally, at low temperatures one expects relaxation processes to bring the system to the ground state with *n*_*kσ*_=0. A peculiarity of superconducting junctions is that internal relaxation processes cannot change the parity of the quasiparticle number. Parity changing processes require a quasiparticle from the bulk, and are therefore rare as the concentration of such quasiparticles is exponentially small at low temperatures. So one can say that a superconducting junction can be in two different ground states, with even or odd parity. The switching between parities occurs on a long time scale: experiments with break junctions^21^, for example, yield switching times >0.1 ms, while for two other recent experimental setups 10 ms (ref. 22) and 1 min (ref. 23) have been reported. We therefore expect a switching of the Chern number and the associated quantized transconductance on this time scale. In particular, for the situation we concentrate on, the nontrivial Chern number *C* comes from the lowest band, and the transconductance would switch between −(4*e*^2^/*h*)*C* for even parity and zero for odd parity. If the current is averaged over time intervals much longer than the switching scale, the resulting transconductance will be proportional to the probability of finding the junction in the even parity state.

Therefore, the topological signature is robust provided the fermion parity is preserved, a fact which has also been pointed out for other topological systems realized in superconductors^9^. There are ways to control quasiparticle poisoning and reach a desired even parity in the ABS occupation^7^,^24^. Note that quantum Hall-like conductance quantization has also been proposed in superconducting devices with finite charging energy and hosting quantum phase slips^25^. Furthermore, superconducting junctions with a gate-tunable charging energy may realize topologically protected discrete charge pumping^26^.

We now focus on a four-terminal junction and investigate the energy spectrum as a function of the three independent phases *ϕ*_1,2,3_. As mentioned above, such a 3D bandstructure may host Weyl points with positive or negative topological charge. The Nielsen-Ninomiya theorem^27^ implies that the total topological charge of the system is zero, such that the number of Weyl points is always even. Furthermore, time-reversal invariance corresponds to a mapping from 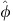 to 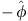, hence a Weyl point at 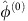 has a counterpart at 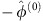 with the same topological charge. Thus, Weyl points come in groups of 4.

In the simplest case, where each contact contains only one channel, the system may realize 0 or 4 Weyl points, corresponding to a topologically trivial or nontrivial 3D material, respectively. If a scattering matrix yielding Weyl points is found, small changes in the scattering matrix only modify their position, but cannot gap them. Namely, as the Weyl points carry a topological charge, individual Weyl points are stable and annihilation is possible only when two Weyl points with opposite charges coincide.

**Figure 2:**
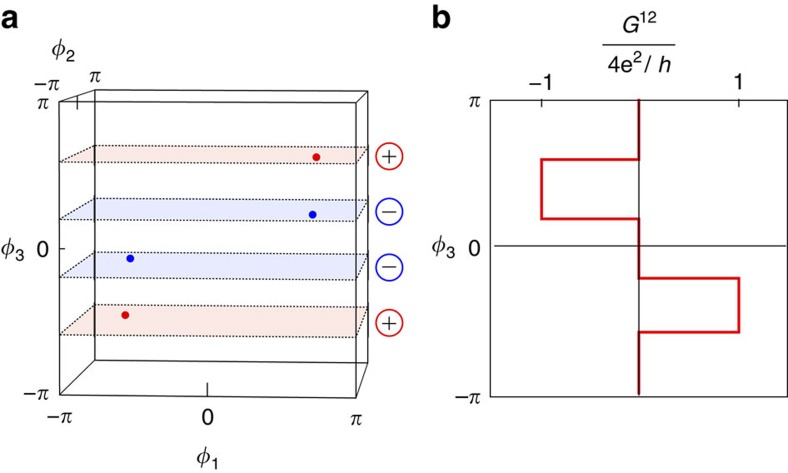
Topological characterization of the 4-terminal junction for the single-channel case. (**a**) Position of the four Weyl points in the space of *ϕ*_1,2,3_ of the single-channel 4-terminal junction, the colour code indicating the respective charge. (**b**) The resulting transconductance *G*^12^ indicating the Chern number, as a function of *ϕ*_3_ for the same single-channel junction as in **a**.

A specific example is shown in Fig. 2. The position and charge of the 4 Weyl points is shown in Fig. 2a. Without loss of generality, we fix the phase *ϕ*_3_ and compute the transconductance *G*^12^ between voltage-biased contacts 1 and 2. In Fig. 2b, one can clearly see that the transconductance increases (decreases) by 4*e*^2^/*h* when *ϕ*_3_ passes through a Weyl point with positive (negative) charge. Interpreting *ϕ*_3_ as a control parameter rather than a quasimomentum, we thus see that the 2D bandstructure of the system as a function of *ϕ*_1_ and *ϕ*_2_ undergoes a topological transition when *ϕ*_3_ passes through a Weyl point. The transconductance directly measures the Chern number characterizing the corresponding 2D topological phase. Note that the transconductance satisfies the relation *G*^12^(−*ϕ*_3_)=−*G*^12^(*ϕ*_3_) due to time-reversal symmetry.

**Figure 3:**
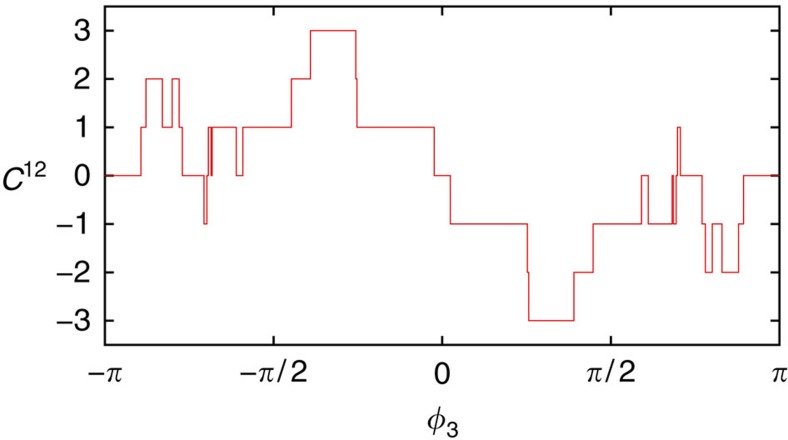
Topological characterization of the 4-terminal junction for the multi-channel case. Chern number as a function of *ϕ*_3_ for a multi-channel 4-terminal junction, where the contacts 1, 2, 3, and 0 contain 12, 11, 10, and 9 channels, respectively. In this particular example, the junction hosts 36 Weyl points.

By randomly generating scattering matrices from the circular orthogonal ensemble^28^, we find that about 5% of scattering matrices give rise to four Weyl points (Supplementary Note 4). More Weyl points can be obtained in multi-channel junctions where the maximal number of Weyl points is roughly proportional to the number of channels, and the probability to have no Weyl points is small (Supplementary Fig. 2). As a consequence, a greater variety of 2D topological phases with higher Chern numbers can be realized in that case. This is shown in Fig. 3 for a multi-channel junction hosting 36 Weyl points, where the maximal Chern number is 3. Recently realized few-channel cross junctions^29^ are promising to observe the transconductance in a four-terminal junction.

We now turn to five-terminal junctions. In that case, the Weyl singularities appear as closed loops in the 4D space of phases. The simplest way to visualize them is to consider the additional phase *ϕ*_4_ as a tuning parameter of the 3D system described by the phases (*ϕ*_1_, *ϕ*_2_, *ϕ*_3_). Tuning *ϕ*_4_ the Weyl points move, but remain at zero energy. Note that in the 3D subspace, time-reversal symmetry is effectively broken, as for a fixed nonzero *ϕ*_4_, a Weyl point at 
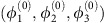
 does not have a counterpart at 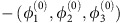 anymore. The only constraint at a finite *ϕ*_4_ is that the number of Weyl points is even. Once two Weyl points with opposite charge meet, they annihilate. Thus their trajectories describe closed loops in the 4D space of all phases.

**Figure 4:**
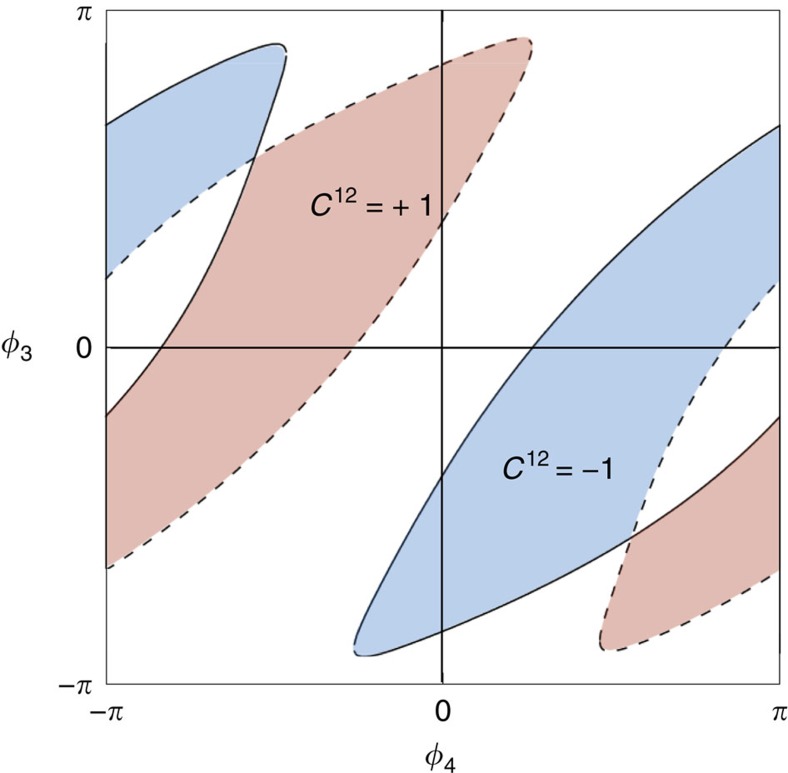
Topological characterization of a 5-terminal junction, each contact having a single channel. The coloured areas display a nonzero Chern number *C*^12^. The boundaries of these areas correspond to the projection of the Weyl singularity lines to the (*ϕ*_3_, *ϕ*_4_)-plane. In a 3D subspace the curves can be assigned a charge.

As before, the presence of Weyl singularities may be probed by the transconductance between two voltage-biased terminals, say terminal 1 and 2. As a function of the two other phases *ϕ*_3_ and *ϕ*_4_, we now obtain areas with different quantized values of the transconductance, corresponding to different Chern numbers. The boundaries of these areas are given by the projections of the Weyl loops on the (*ϕ*_3_, *ϕ*_4_)-plane. An example is shown in Fig. 4. Here time-reversal symmetry of the scattering matrix manifests itself in the relation *G*^12^(−*ϕ*_3_, −*ϕ*_4_)=−*G*^12^(*ϕ*_3_, *ϕ*_4_).

## Discussion

The above considerations are straightforwardly extended to a larger number of terminals. Furthermore, as we saw for the five-terminal junction, breaking time-reversal invariance does not lift the topological protection of the Weyl points at zero energy, as long as spin degeneracy is preserved. When spin degeneracy is lifted due to a Zeeman field or due to spin–orbit interactions, we expect the Weyl points to shift away from zero energy while remaining stable (Supplementary Note 5 and Supplementary Fig. 3). The possibility of realizing zero-energy states in three-terminal junctions with strong spin–orbit interaction was studied in ref. 30.

To summarize, we predict the existence of topological Weyl singularities in the ABS spectrum of *n*-terminal superconducting junctions with *n*≥4. These Weyl singularities manifest themselves in a quantized transconductance between two voltage-biased contacts, when the remaining phases are tuned away from the singularities. Tuning the system through a Weyl singularity, the conductance displays a step, signalling a topological transition of the 2D system described by the two phases of the voltage-biased contacts. This signature is robust provided the system remains in its ground state. Thus, the quantized transconductance should be accessible experimentally at low temperatures and voltages, in multi-terminal junctions with, for example, 2D electron gases, semiconducing crossed nanowires or graphene (all systems through which a conventional Josephson effect has already been measured).

## Additional information

**How to cite this article:** Riwar, R.-P. *et al*. Multi-terminal Josephson junctions as topological matter. *Nat. Commun.* 7:11167 doi: 10.1038/ncomms11167 (2016).

## Supplementary Material

Supplementary Information Supplementary Figures 1-3, Supplementary Notes 1-5 and Supplementary References
