# Supplementary material for: Multi-terminal Josephson junctions as topological matter
Source: Nat Commun. 2016 Apr 4;7:11167. doi: 10.1038/ncomms11167 (PMC4822011; doi:10.1038/ncomms11167)
Supplement: Supplementary Information — Supplementary Figures 1-3, Supplementary Notes 1-5 and Supplementary References [file ncomms11167-s1.pdf]

Supplementary Note 1

Derivation of the Weyl Hamiltonian

In the following, we provide the derivation of the Weyl Hamiltonian, Eq. (2) of the main text, and associated properties of the Andreev bound state (ABS) spectrum.

The topological properties of multi-terminal junctions result from the appearance of Weyl singularities in the ABS spectrum. At subgap energies, $|E| < \Delta$, there are no propagating states in the superconducting leads. However, the interplay of Andreev reflection and electron scattering through the constriction results in the formation of a discrete spectrum of bound states. The ABS for an n-terminal superconducting junction are determined through the condition of having a unity eigenvalue [1]

$$|\psi\rangle = \hat{S}_N \hat{S}_A |\psi\rangle \,, \quad \text{with} \quad \hat{S}_N = \begin{pmatrix} \hat{S} & 0 \\ 0 & \hat{S}^* \end{pmatrix} \quad \text{and} \quad \hat{S}_A = \begin{pmatrix} 0 & e^{i\hat{\phi}} \\ e^{-i\hat{\phi}} & 0 \end{pmatrix} e^{-i\chi} \,, \tag{1}$$

with $|\psi\rangle$ being the corresponding eigenvector. Here, the two-by-two structure indicates the Nambu space of electrons and holes. The scattering matrix $\hat{S}_N$ describes the normal metal scattering region, where $\hat{S}$ and $\hat{S}^*$ provide the scattering amplitudes for electrons and holes, respectively. The matrix $\hat{S}_A$ accounts for the phase acquired in the Andreev reflection processes, where $\chi = \arccos(E/\Delta)$ and $e^{i\hat{\phi}}$ is a diagonal matrix that assigns to each channel the phase of the corresponding terminal. Note that the wave function $|\psi\rangle$ is written in terms of the outgoing states in a given spin sector $\sigma =\uparrow, \downarrow$.

To determine the ABS energy bands, it is sufficient to reduce the above equation to a determinant condition for the bound state energies E, $\det(1 - \hat{S}_N \hat{S}_A) = 0$. Reducing that equation to the electron subspace, we find

$$\det\left[1 - e^{-2i\chi} \hat{A}(\hat{\phi})\right] = 0 \,, \tag{2}$$

with the unitary matrix $\hat{A}(\hat{\phi}) = \hat{S} e^{i\hat{\phi}} \hat{S}^* e^{-i\hat{\phi}}$. The matrix $\hat{A}$ possesses the particle-hole symmetry $\hat{A}^* = \hat{U}^\dagger \hat{A} \hat{U}$, where $\hat{U}(\hat{\phi}) = e^{i\hat{\phi}} \hat{S}^T$. This implies that the eigenvalues of $\hat{A}$ come in pairs, $e^{\pm i a_k}$,

corresponding to energies $\pm E_k$, where $E_k = \Delta\cos(a_k/2)$ with $0 \leq a_k \leq \pi$. For an odd number of channels, there is an additional eigenvalue 1 corresponding to a state at the gap edge. We assign eigenvectors $|\Psi_k^+\rangle$ and $|\Psi_k^-\rangle = \hat{U}|\Psi_k^+\rangle^*$ to the pair of eigenvalues $e^{\pm i\alpha_k}$, respectively.

If there is a zero-energy solution at $\hat{\phi}^{(0)}$, the spectrum of the unitary matrix $\hat{A}(\hat{\phi}^{(0)})$ has a doubly degenerate eigenvalue -1. The corresponding orthogonal eigenvectors are given as $|a^+\rangle = |\Psi_0^+(\hat{\phi}^{(0)})\rangle$ and $|a^-\rangle = |\Psi_0^-(\hat{\phi}^{(0)})\rangle$. In the vicinity of the singularity at $\hat{\phi}^{(0)}$, we expand the determinant equation, Supplementary Equation 2, for small $\delta\hat{\phi} = \hat{\phi} - \hat{\phi}^{(0)} \ll 1$ and $E \ll \Delta$. Up to first order, we find $e^{i2\chi} \approx -1 + 2iE/\Delta$ and

$$\hat{A}(\hat{\phi}) \approx \hat{A}(\hat{\phi}^{(0)}) + i\hat{S}\,\delta\hat{\phi}\hat{S}^\dagger\hat{A}(\hat{\phi}^{(0)}) - i\hat{A}(\hat{\phi}^{(0)})\delta\hat{\phi}\,. \tag{3}$$

Projecting Supplementary Equation 2 onto the subspace defined by $|a^+\rangle$ and $|a^-\rangle$, and keeping only the lowest-order terms, we find the determinant equation

$$\det\left[E - \hat{H}_\mathrm{W}\right] = 0\,. \tag{4}$$

It defines the eigenvalue problem for the lowest band, described by the two-by-two Weyl Hamiltonian,

$$\hat{H}_\mathrm{W} = \frac{\Delta}{2}\begin{pmatrix} \langle a^+|\,\delta\hat{\phi} - \hat{S}\,\delta\hat{\phi}\hat{S}^\dagger\,|a^+\rangle & \langle a^+|\,\delta\hat{\phi} - \hat{S}\,\delta\hat{\phi}\hat{S}^\dagger\,|a^-\rangle \\ \langle a^-|\,\delta\hat{\phi} - \hat{S}\,\delta\hat{\phi}\hat{S}^\dagger\,|a^+\rangle & \langle a^-|\,\delta\hat{\phi} - \hat{S}\,\delta\hat{\phi}\hat{S}^\dagger\,|a^-\rangle \end{pmatrix}\,. \tag{5}$$

Using the particle-hole symmetry $|a^-\rangle = \hat{U}(\hat{\phi}^{(0)})|a^+\rangle^*$, Supplementary Equation 5 may be simplified to

$$\hat{H}_\mathrm{W} = \sum_{i=x,y,z} h_i\,\hat{\tau}_i\,, \tag{6}$$

where $\hat{\tau}_{x,y,z}$ are Pauli matrices in the basis of $\{|a^+\rangle, |a^-\rangle\}$, and the real prefactors h_i take the form $h_x + ih_y = \Delta\langle\delta\hat{\phi}\rangle_{-+}$ and $h_z = \frac{\Delta}{2}\left(\langle\delta\hat{\phi}\rangle_{++} - \langle\delta\hat{\phi}\rangle_{--}\right)$. As a consequence, the energy spectrum is linear in $\delta\hat{\phi}$ and reads $E = \pm\sqrt{h_x^2 + h_y^2 + h_z^2}$. We see that 3 independent parameters are necessary to tune the energy to zero. Thus, the Weyl singularities in general appear as points in

the space of three independent phases of the 4-terminal junction, and as curves in the space of four independent phases of the 5-terminal junction.

In a given 3D subspace, one may assign a charge to each Weyl point. The sign of a Weyl charge is most conveniently defined by rewriting the Weyl Hamiltonian in matrix form, $\hat{H}_{\mathrm{W}} = \sum_{\alpha i} \delta\phi_\alpha M_{\alpha i} \hat{\tau}_i$ with $M_{\alpha i} = \partial_{\delta\phi_\alpha} h_i$. Then, in a subspace of three independent phases, $\hat{M}$ is a square matrix, and the sign of the charge is given by the sign of the determinant, $\det[\hat{M}]$.

While particle-hole symmetry is local in $\hat{\phi}$, time-reversal symmetry links $\hat{\phi}$ and $-\hat{\phi}$. As a consequence, a junction with time-reversal symmetry gives rise to pairs of Weyl singularities at $\hat{\phi}^{(0)}$ and $-\hat{\phi}^{(0)}$. Indeed, using $\hat{S}^T = \hat{S}$, we find the relation $\hat{A}(-\hat{\phi}) = e^{-i\hat{\phi}}\hat{A}^\dagger(\hat{\phi})e^{i\hat{\phi}}$. Using this relation, we find that the Weyl Hamiltonians near $\hat{\phi}^{(0)}$ and $-\hat{\phi}^{(0)}$ take the same form and hence, in a given 3D subspace, the corresponding Weyl points have the same charge.

We note that there is the possibility of Weyl singularities at finite energy. Due to particle-hole symmetry, they appear in pairs at energies $\pm E$ with the same charge. Because of particle-hole symmetry and spin degeneracy, solutions at energy $-E$ can be ascribed to solutions at energy E in the opposite spin sector. Thus, zero-energy Weyl points are singly degenerate, while finite energy Weyl points are doubly degenerate. As the finite energy Weyl singularities do not affect the ground state Chern numbers used to characterize the system, we do not discuss them any further. Note that the outgoing wavefunctions in particle-hole space used to compute the Chern numbers are given as $|\psi_k^\pm\rangle = (|\Psi_k^\pm\rangle, e^{-i\chi}\hat{U}^\dagger|\Psi_k^\pm\rangle)^T$. Furthermore, $|\psi_{k\sigma}\rangle$ is identified with $|\psi_k^+\rangle$ in the corresponding spin sector σ.

Supplementary Note 2

2.1 Derivation of the current

In the following, we establish the connection between the current and the Berry curvature, and discuss the conditions for adiabaticity.

The current operator through lead α is defined as

$$\hat{I}_\alpha = \frac{2e}{\hbar}\frac{\partial \hat{H}}{\partial \phi_\alpha}\,, \tag{7}$$

where $\hat{H}$ is the Bogoliubov-de Gennes Hamiltonian. In order to calculate its expectation value for time-dependent phases $\hat{\phi}(t)$, we introduce the basis of instantaneous wave functions of the time-dependent Bogoliubov-de Gennes Hamiltonian $\hat{H}(t)$, such that $E_k(t)|\varphi_{k\sigma}(t)\rangle = \hat{H}(t)|\varphi_{k\sigma}(t)\rangle$. Solving the time-dependent equation $i\hbar|\dot{\varphi}\rangle = \hat{H}(t)|\varphi\rangle$ in that basis, up to first order in phase velocity $\dot{\phi}$, we obtain the current contribution from state k with spin σ as

$$I_{\alpha k}(t) \approx 2e\left[\frac{1}{\hbar}\frac{\partial E_k(t)}{\partial \phi_\alpha} - i\frac{\partial\,\langle\varphi_{k\sigma}(t)|}{\partial \phi_\alpha}|\dot{\varphi}_{k\sigma}(t)\rangle + i\langle\dot{\varphi}_{k\sigma}(t)|\frac{\partial\,|\varphi_{k\sigma}(t)\rangle}{\partial \phi_\alpha}\right]\,. \tag{8}$$

Note that $I_{\alpha k}$ does not depend on spin. The first term corresponds to the adiabatic supercurrent. Introducing the Berry curvature $B_k^{\alpha\beta} = -2\mathrm{Im}\left[\partial_{\phi_\alpha}\langle\varphi_{k\sigma}|\partial_{\phi_\beta}|\varphi_{k\sigma}\rangle\right]$, the second term reads $-2e\sum_\beta \dot{\phi}_\beta B_k^{\alpha\beta}$. The many-body expectation value of the current is computed as

$$I_\alpha(t) = \sum_{k\sigma} I_{\alpha k}(t)\left[n_{k\sigma} - \frac{1}{2}\right]\,, \tag{9}$$

where $n_{k\sigma} = 0, 1$ is the occupation of the ABS k with spin σ.

Supplementary Equations 8 and 9 can be used to compute the adiabatic correction to the current, if the energy spectrum is discrete and the occupations of each state do not change with time. For a few-channel junction, the level spacing between the ABS scales as $\sim \Delta$, thus providing the adiabaticity condition $\hbar\dot{\phi} \ll \Delta$. We then may focus on the ground state of the system, where all ABS are empty.

On the other hand, defining the current contribution of the states in the continuum above the gap, as well as of ABS that reach the gap edge at some values of the phases (see the following section in the same Supplementary Note), is problematic. In that case, the Chern number of these states is not well-defined. In the following, we argue that those states do not contribute to the topological properties considered in our work.

Indeed, multi-terminal junctions are known to be topologically trivial in certain cases. For instance, let us consider the case where terminals α and β are voltage biased. When all the other phases ϕ_γ ($\gamma \neq \alpha, \beta$) are set to zero, the junction is effectively a 3-terminal junction which is topologically trivial. In that case, the associated Chern number $C^{\alpha\beta}$ vanishes. A change of $C^{\alpha\beta}$, corresponding to a topological transition, may occur when a state crosses the Fermi level as the phases ϕ_γ are varied. As the energy spectrum is gapped, this can only occur for the lowest energy states. Thus, only those states contribute to the topological properties, and in the following we only retain their contribution in Eqs. 8 and 9. In particular, we keep the ABS states that cross at $E = 0$, as well as all other states that may cross those states at finite energy.

This establishes the regime of validity for the relation between the transconductance and Chern number given in the main text, see Eq. (5). Namely, only the gap of the lowest ABS matters, thus limiting the phase velocity to $\hbar\dot{\phi} \ll \Delta$, as stated above. To compute the Chern numbers, we use the outgoing wavefunctions $|\psi_{k\sigma}\rangle$ defined in the previous section. Note that the Chern number may be computed using either the outgoing or the incoming wave functions as they are related through $\hat{S}_N$ which does not depend on the superconducting phases.

2.2 Comment on Andreev bound states reaching the gap edge

Let us return to the possibility of the ABS with the highest energy reaching the gap edge. Indeed we find that, for a system with an even number of total channels, this occurs along $(n-1)$-dimensional subspaces in the space of n independent phases. As pointed out previously, in the case of an odd number of total channels, there is a state at Δ for any value of the phases.

An argument for this fact can be given in analogy with the derivation of the Weyl Hamiltonian. Namely, an ABS reaching the gap edge at some $\hat{\phi}_\mathrm{g}$ requires that the unitary matrix $A(\hat{\phi}_\mathrm{g})$ has a doubly degenerate eigenvalue $+1$. We denote the corresponding orthogonal eigenvectors $|b^+\rangle$ and $|b^-\rangle = \hat{U}(\hat{\phi}_g)|b^+\rangle^*$. Expanding Supplementary Equation 2 for small $\delta\hat{\phi} = \hat{\phi} - \hat{\phi}_\mathrm{g}$, we

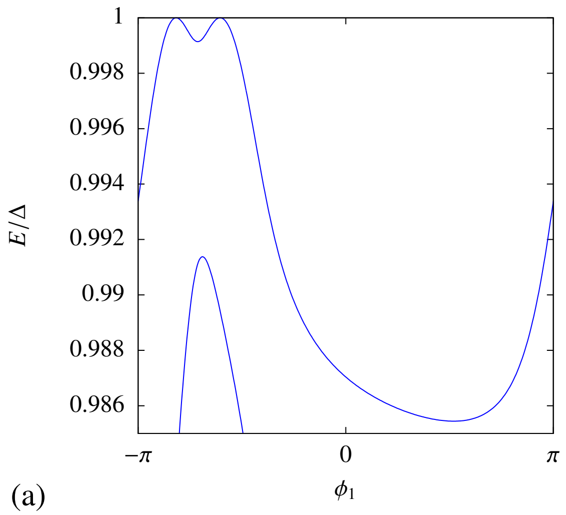

(a)

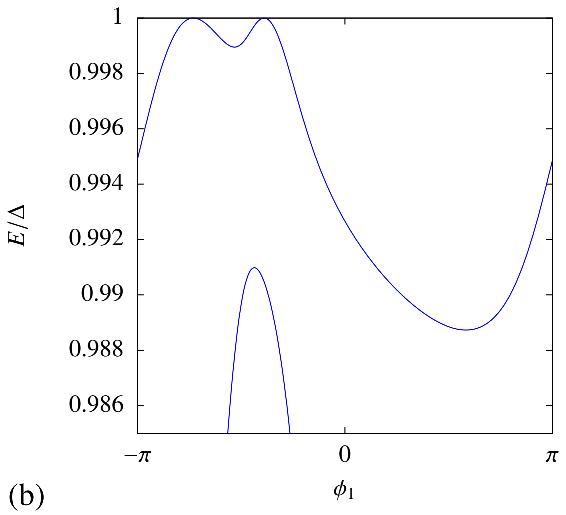

(b)

Supplementary Figure 1: **Gap edge touching of the highest ABS of the spectra** Panels a and b show the zoom of Figs. 1b and 1c in the main text, respectively. The gap edge touchings are clearly visible.

can reduce the determinant equation to

$$\det\left[\operatorname{sign}(E)\sqrt{\frac{8(\Delta-|E|)}{\Delta}}+\left(\langle\delta\hat{\phi}\rangle_{++}-\langle\delta\hat{\phi}\rangle_{--}\right)\hat{\tau}'_z\right]=0\,, \tag{10}$$

where $\hat{\tau}'_z$ is a Pauli matrix in the basis $\{|b^+\rangle, |b^-\rangle\}$.

Thus, the solution for the ABS energies reads

$$E\approx\pm\Delta\left[1-\frac{1}{8}\left(\langle\delta\hat{\phi}\rangle_{++}-\langle\delta\hat{\phi}\rangle_{--}\right)^2\right]. \tag{11}$$

Furthermore, we see that the ABS wave function has a discontinuity at $\hat{\phi}_{\mathrm{g}}$. Namely, the wave function of the state at positive energy is given by $|b^+\rangle$ for $\langle\delta\hat{\phi}\rangle_{++}-\langle\delta\hat{\phi}\rangle_{--}<0$ and by $|b^-\rangle$ for $\langle\delta\hat{\phi}\rangle_{++}-\langle\delta\hat{\phi}\rangle_{--}>0$, and vice versa for the states at negative energies.

In contrast to the discussion for the zero-energy Weyl points, here a single parameter is sufficient to tune the ABS energy to the gap edge. Therefore, the highest ABS reaches the gap edge along $(n-1)$-dimensional subspaces in the space of n independent phases. This is visible in Figs. 1b and 1c in the main text. A zoom is shown in Supplementary Figure 1.

When sweeping two phases, the curves where the highest ABS reaches the continuum cannot be avoided. If this state was occupied, this would break adiabaticity even for arbitrary slow driving. (Introducing a finite energy-dependence of the scattering matrix or spin-orbit interactions may create a finite level repulsion between the highest ABS and the continuum and, thus, restore adiabaticity, possibly under stricter conditions. This would need further investigation.) However, as far as the topological characterization of the junction is concerned, we came to the conclusion that this peculiarity should not change our results, see previous section in this Supplementary Note. A possible exception could be the case where an additional Weyl point at finite energy involving the highest ABS occurs, which we did not observe.

Supplementary Note 3

Estimation of the current noise

The topological signal of interest is a small dc contribution on top of a large ac adiabatic current. This makes it important to provide an estimate for the measurement time T_0 required to sufficiently average the current signal. In particular, the lower bound for T_0 is provided by $S_I/|\bar{I}|^2$, where $\bar{I}$ denotes the dc current signal and S_I is the low-frequency current noise. The dominant contribution to S_I stems from fluctuations of the phases due to voltage noise and will be estimated in the following.

We define the current noise as

$$S_I = \int d\tau \, \langle I(t) I(t+\tau) \rangle \ , \tag{12}$$

where the brackets $\langle \ldots \rangle$ denote the expectation value with respect to the statistical ensemble. As the adiabatic supercurrent, i.e., the first term in Supplementary Equation 9, is much larger than the correction proportional to the Berry phase, it dominates the noise. We exploit the fact that this current is periodic in both phases ϕ_α and ϕ_β, such that it may be expanded in Fourier

harmonics,

$$I(\phi_\alpha, \phi_\beta) = \sum_{n_\alpha, n_\beta=-\infty}^{\infty} I_{n_\alpha, n_\beta} e^{i(n_\alpha \phi_\alpha + n_\beta \phi_\beta)} . \tag{13}$$

Substituting Supplementary Equation 13 into Supplementary Equation 12, we obtain

$$S_I = \int d\tau \sum_{n_\alpha, n_\beta} |I_{n_\alpha, n_\beta}|^2 \langle e^{in_\alpha[\phi_\alpha(t) - \phi_\alpha(t+\tau)]} \rangle \langle e^{in_\beta[\phi_\beta(t) - \phi_\beta(t+\tau)]} \rangle , \tag{14}$$

as the two phases fluctuate independently and the ac contribution averages to zero.

In order to evaluate the averages, we assume white noise in the voltage sources, described by

$$\frac{2e^2}{\hbar^2} \langle \delta V_\alpha(t) \delta V_\alpha(t') \rangle = \Gamma_\alpha \delta(t - t') , \tag{15}$$

with the rate Γ_α associated to the voltage noise in lead α. Using $\phi_\alpha(t + \tau) - \phi_\alpha(t) = \omega_\alpha \tau + (2e/\hbar) \int_t^{t+\tau} ds\, \delta V_\alpha(s)$, where $\omega_\alpha \equiv 2eV_\alpha/\hbar$, we find

$$\langle e^{in_\alpha[\phi_\alpha(t) - \phi_\alpha(t+\tau)]} \rangle = e^{-in_\alpha \omega_\alpha \tau - \Gamma_\alpha n_\alpha^2 |\tau|} , \tag{16}$$

and subsequently

$$S_I = 2 \sum_{n_\alpha, n_\beta} |I_{n_\alpha, n_\beta}|^2 \frac{\Gamma_\alpha n_\alpha^2 + \Gamma_\beta n_\beta^2}{(n_\alpha \omega_\alpha + n_\beta \omega_\beta)^2 + (\Gamma_\alpha n_\alpha^2 + \Gamma_\beta n_\beta^2)^2} . \tag{17}$$

We see that S_I depends on frequencies $\omega \sim \omega_{\alpha,\beta}$ in a quite complex fashion. In the limit of low voltage noise, $\omega \gg \Gamma$ with $\Gamma \sim \Gamma_{\alpha,\beta}$, we have to distinguish between commensurate and incommensurate voltages $V_{\alpha,\beta}$. In the commensurate case, the first term in the denominator of Supplementary Equation 17 vanishes for a pair (n_α, n_β), yielding $S_I \propto 1/\Gamma$. By contrast, in the incommensurate case, the first term in the denominator of Supplementary Equation 17 dominates, yielding a much weaker noise, $S_I \propto \Gamma/\omega^2$. Thus, the current noise strongly varies with ω. Averaging over a window of width $\sim \omega$ yields the average noise $\overline{S_I} \propto 1/\omega$. On the other hand, in the noisy regime $\omega \ll \Gamma$, we find that $S_I \propto 1/\Gamma$, irrespective of the voltages being commensurate or not. Thus, the current noise is actually weaker than the average current noise

in the opposite regime. Furthermore, incommensurability is no longer required for averaging, as the strong phase fluctuations take care of covering the entire unit cell.

This brings us to the conclusion that the strong voltage noise regime $\omega \ll \Gamma$ may be favourable for averaging – provided, of course, that the noise is still in the limit $\hbar\Gamma \ll \Delta$. To estimate the measurement time, we use $I_{n_\alpha,n_\beta} \sim e\Delta/\hbar$ and $\bar{I} \sim e^2 V/\hbar$ with $V \sim V_{\alpha,\beta}$, together with the estimate for S_I found above, to obtain

$$T_0 > \frac{\Delta^2}{(eV)^2}\frac{1}{\Gamma}\,. \tag{18}$$

Supplementary Note 4

Occurence of Weyl points for 4-terminal junctions

Here, we estimate how often random scattering matrices give rise to Weyl points at zero energy.

As discussed in the first section of the supplementary, such Weyl singularities emerge when the matrix A has the eigenvalue -1. Whether this occurs indeed depends on the properties of the junction, encoded in the scattering matrix $\hat{S}$. We performed a numerical analysis for 4-terminal junctions, both in the single-channel and multi-channel cases. For this purpose, we generated symmetric scattering matrices according to the circular orthogonal ensemble using the following recipe [2]: We randomly generated hermitian matrices $\hat{\mathcal{H}}$ from a Gaussian ensemble, and numerically diagonalized them, $\hat{\mathcal{H}} = \hat{\mathcal{U}}^\dagger \hat{\mathcal{D}} \hat{\mathcal{U}}$, where $\hat{\mathcal{U}}$ is a unitary matrix and $\hat{\mathcal{D}}$ is a real diagonal matrix. A random symmetric scattering matrix was then generated as $\hat{S} = \hat{\mathcal{U}}^T \hat{\mathcal{U}}$. For each $\hat{S}$, we numerically checked the existence of zeros of the function $\left|\det[1 + \hat{A}(\hat{\phi})]\right|$ in the space of phases.

In the single-channel case, we ran the check for 965 randomly generated matrices $\hat{S}$, out of which 46 gave rise to zero-energy solutions. Thus, we found that a total of roughly 5% of all scattering matrices yield Weyl points, while the remaining 95% provide a trivial junction.

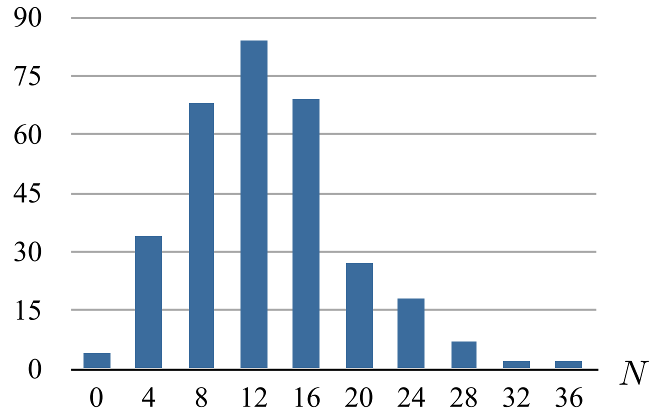

Supplementary Figure 2: **Occurence of topologically nontrivial scattering matrices** Histogram displaying the occurrence of random scattering matrices yielding N Weyl points for the four-terminal multi-channel junction.

When increasing the number of channels in each terminal, we found that the maximal number of Weyl points scales with the number of channels, and that the probability of a junction without Weyl points decreases significantly. A total of 324 random scattering matrices were generated for a junction with four terminals, where the terminals have 12,11,10, and 9 channels, respectively. In Supplementary Figure 2 we show the histogram displaying the occurrence of randomly generated scattering matrices that provide N zero-energy Weyl points. Only 4 scattering matrices gave rise to a junction without zero-energy Weyl points. Note that our algorithm has a small, but finite probability to miss some zeros. As a consequence, while the number of Weyl points has to be a multiple of 4 because of time-reversal symmetry (see main text), the algorithm misidentifies a few cases as having an odd multiple of 2 Weyl points.

Supplementary Note 5

Comment on the effect of broken spin-rotation symmetry

Note that our result is robust with respect to lifting spin degeneracy due to a Zeeman field or spin-orbit coupling. This is most easily explained in the case of a Zeeman field which simply

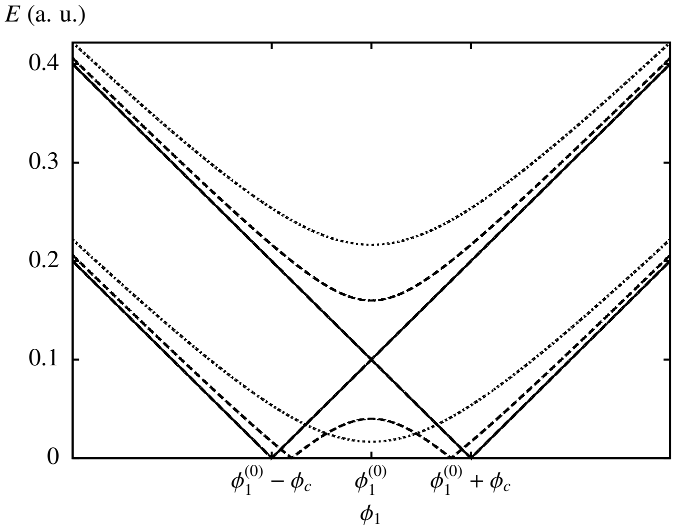

Supplementary Figure 3: **Weyl point at finite energy due to splitting of spin bands** We show qualitatively cross sections along ϕ_1 of the lowest ABS of a four terminal junction when the two spin bands are split. The position of the Weyl point is at $(\phi_1^{(0)}, \phi_2^{(0)}, \phi_3^{(0)})$. We set $\phi_2 = \phi_2^{(0)}$. For $\phi_3 = \phi_3^{(0)}$ the cross section passes the Weyl point at finite energy (solid line), for $|\phi_3 - \phi_3^{(0)}| < \phi_c$ it passes through the surface of zero energy solutions (dashed line), while for $|\phi_3 - \phi_3^{(0)}| > \phi_c$ the spectrum is again fully gapped (dotted line), and the transconductance is quantized. The parameter ϕ_c denotes the radius of the zero energy surface and depends on the magnitude of the spin splitting.

shifts the up- and down spin bands in opposite directions. Thus, the Weyl point is moved to finite energy while zero-energy states exist on a surface enclosing the Weyl point, see Supplementary Figure 3. If the plane swept by the phases of the voltage-biased terminals intersects this surface of $E = 0$, the transconductance is still quantized in a given parity sector.

To understand the role of spin-orbit coupling, we note that, as has been shown in Refs. [3, 4], it also leads to a splitting between bands in multi-terminal junctions. In fact, locally it acts the same way as a Zeeman field. Then, using the same arguments as above, we conclude that for sufficiently weak spin-orbit coupling the transconductance quantization is conserved.

Supplementary References

[1] C. W. J. Beenakker, Universal limit of critical-current fluctuations in mesoscopic Josephson junctions, Phys. Rev. Lett. **67**, 3836 (1991).

[2] M. L. Mehta, Random matrices, vol. 142 (Elsevier/Academic Press, Amsterdam, 2004).

[3] N. M. Chtchelkatchev and Yu. V. Nazarov, Andreev quantum dots for spin manipulation, Phys. Rev. Lett. **90**, 226806 (2003).

[4] B. van Heck, S. Mi, and A. R. Akhmerov, Single fermion manipulation via superconducting phase differences in multiterminal Josephson junctions, Phys. Rev. B **90**, 155450 (2014).
